# Fly DPP10 acts as a channel ancillary subunit and possesses peptidase activity

**DOI:** 10.1038/srep26290

**Published:** 2016-05-20

**Authors:** Yohei Shiina, Tomohiro Muto, Zhili Zhang, Ahmad Baihaqie, Takamasa Yoshizawa, Hye-in J. Lee, Eulsoon Park, Shinya Tsukiji, Koichi Takimoto

**Affiliations:** 1 Department of Bioengineering, Nagaoka University of Technology, 1603-1 Kamitomioka, Nagaoka, Niigata 940-2188, Japan; 2 Frontier Research Institute for Materials Science, Nagoya Institute of Technology, Gokiso-cho, Showa-ku, Nagoya 466-855, Japan; 3 Department of Materials Science and Engineering, Nagoya Institute of Technology, Gokiso-cho, Showa-ku, Nagoya 466-855, Japan

## Abstract

Mammalian DPP6 (DPPX) and DPP10 (DPPY) belong to a family of dipeptidyl peptidases, but lack enzyme activity. Instead, these proteins form complexes with voltage-gated K^+^ channels in Kv4 family to control their gating and other properties. Here, we find that the fly DPP10 ortholog acts as an ancillary subunit of Kv4 channels and digests peptides. Similarly to mammalian DPP10, the fly ortholog tightly binds to rat Kv4.3 protein. The association causes negative shifts in voltage dependence of channel activation and steady state inactivation. It also results in faster inactivation and recovery from inactivation. In addition to its channel regulatory role, fly DPP10 exhibits significant dipeptidyl peptidase activity with Gly-Pro-MCA (glycyl-L-proline 4-methylcoumaryl-7-amide) as a substrate. Heterologously expressed Flag-tagged fly DPP10 and human DPP4 show similar *K*m values towards this substrate. However, fly DPP10 exhibits approximately a 6-times-lower relative *k*_cat_ value normalized with anti-Flag immunoreactivity than human DPP4. These results demonstrate that fly DPP10 is a dual functional protein, controlling Kv4 channel gating and removing bioactive peptides.

DPP4-related proteins constitute a small group of dipeptidyl peptidases that are capable of hydrolyzing a prolyl bond two residues from the N-terminus. The group includes DPP4, FAP (fibroblast activation protein), DPP8 and DPP9^1^,^2^,^3^. Since these enzymes potentially degrade important hormones, cytokines and other proteins, they have attracted much interest as pharmacological targets for treatment of metabolic, immune and other disorders^4^,^5^,^6^. In contrast to these enzymes, the two structurally-related members DPP6 (DPPX) and DPP10 (DPPY) lack peptidase activity^7^,^8^. Indeed, the corresponding polypeptides contain aspartic acid and glycine, respectively, at the position of the catalytic serine in DPP4-related enzymes. Instead, these two proteins tightly bind to pore-forming subunits of voltage-gated K^+^ channels in Kv4 family^9^,^10^,^11^,^12^. The channel complexes containing DPP6 or DPP10 display various gating properties that resemble fast-inactivating A-type K^+^ currents in native neurons^13^,^14^. Thus, mammalian DPP6 and DPP10 act as auxiliary subunits for neuronal Kv4 channels.

Crystal structures of mammalian DPP4, DPP6 and DPP10 reveal the common structural organization in their extracellular regions with a unique eight-blade β propeller and α/β-hydrolase fold^15^,^16^,^17^. The structural studies also indicated that the lack of enzymatic activity in mammalian DPP6 and DPP10 was not simply the result of the amino acid substitutions at the catalytic serine^16^,^17^. The positions of several other residues and functional groups in the catalytic triad and substrate-recognizing portion show notable differences between the two non-enzyme proteins and DPP4. Thus, mammalian DPP6 and DPP10 might have lost various structural features required for peptidase activity during evolution. Moreover, the transmembrane and its surrounding region of DPP6 and DPP10 polypeptides show significant sequence divergence from the corresponding portion of DPP4 protein. Our previous work demonstrated that this region of the two non-enzyme proteins mediate specific binding to Kv4 proteins^11^. These findings indicate that mammalian DPP6 and DPP10 act solely as auxiliary subunits of Kv4 channels, but not dipeptidyl peptidase.

Channel ancillary subunits might arise from proteins with other functional roles during evolution. In some cases, these proteins may retain the original or related function while gaining a new channel-regulatory role. Furthermore, the original or related functions may control gating and other properties of the associated channel. For instance, Kvβ proteins are functional oxidoreductases that act as Kv1-channel ancillary subunits^18^,^19^. Mutations in the catalytic or NADPH-binding sites, as well as the cofactor binding itself, influence the ability of Kvβ subunit to alter channel inactivation^20^,^21^,^22^. Moreover, oxidation of NADPH on Kvβ has been shown to influence inactivation of the associated channel^23^. Thus, Kvβ subunit may act as a redox sensor to control excitability. We wondered if any channel-interacting peptidase homologs might retain enzymatic activity, potentially acting as a link between the enzymatic function to excitability. Inspection of primary structures of dipeptidyl peptidases and their homologs in the GenBank detected potential dual functional molecules in non-mammalian species. In this paper, we show that the fly DPP10 ortholog possesses a dual function as channel-ancillary subunit and peptidase.

## Results

### Primary structure of fly DPP10

**Figure 1:**
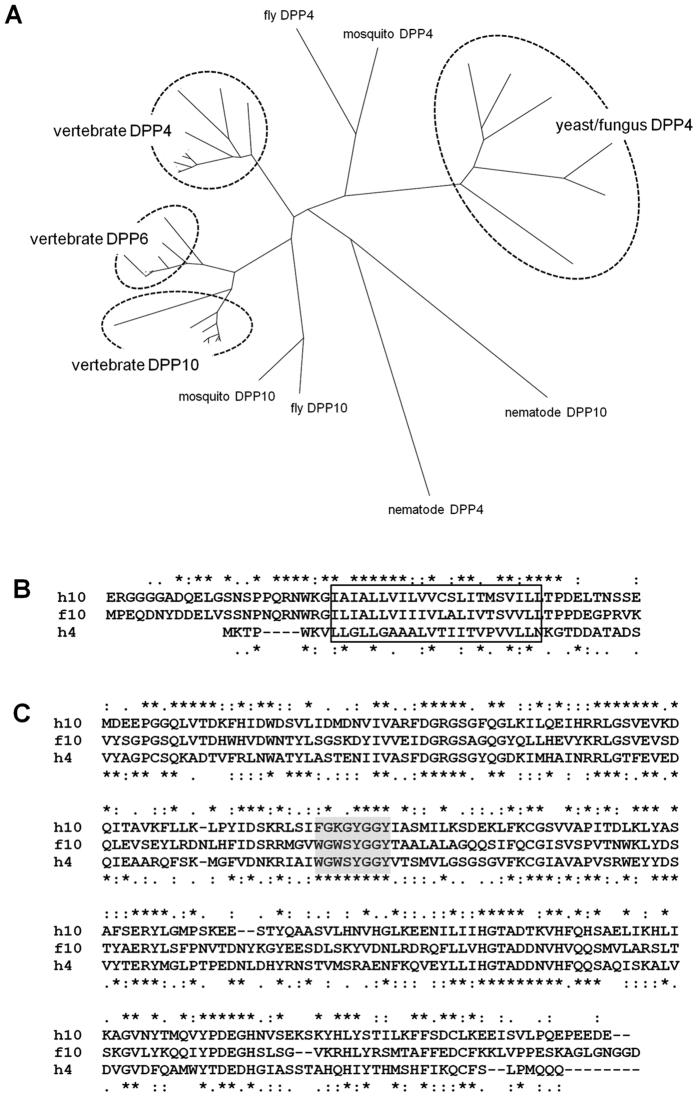
Primary structures of fly DPP10 and related proteins. (**A**) A phylogenetic tree was constructed with DPP4, DPP10 and DPP6 orthologs in various species using an unrooted neighbor-joining method. The partial and full sequence data of *Anopheles gambiae* (XP_314886 and XP_317288) were used for mosquito DPP10 and DPP4, respectively. Note that nematode DPP4 and DPP10 (*Caenorhabditis elegans* NP_506850 and NP_510461) are positioned at a same branch in this tree. Yet, the transmembrane region of nematode DPP10 shows high similarity to DPP10 in other species, suggesting that nDPP10 may act as an auxiliary subunit for Kv4 channel. (**B,C**) DPP10 and DPP4 comprise similar domain organizations. The amino acid sequences of human DPP10 (hDPP10), fly DPP10 (fDPP10), and human DPP4 (hDPP4) are aligned for the transmembrane portion (**B**) and peptidase domain (**C**). Marks (*, :,.) indicate identical, highly similar, and moderately similar amino acids, respectively, between fDPP10 and hDPP10 (above), and between fDPP10 and hDPP4 (below). A square in (**A**) indicates the predicted membrane-spanning region. The portion encompassing the catalytic serine in (**B**) is shadowed.

We searched for the presence of DPP6 and DPP10 orthologs in various species using HomoloGene (NCBI). DPP10 orthologs were found in a wide range of animal species in the criterion named *Bilateria*, whereas DPP6 counterparts were only in vertebrates. In addition, DPP4 orthologs were seen in an even wider range of eukaryotes including plants and yeast. A phylogenetic tree constructed with these DPP sequences in various species indicates that fly and mosquito DPP10s are diverged from the group including vertebrate DPP6 and DPP10 (Fig. 1A). Likewise, insect DPP4s are placed between vertebrate and yeast/fungus DPP4s. Thus, insects and some other animal species may contain only one gene for channel-regulatory dipeptidyl peptidase homolog.

The fly DPP10 polypeptide (GenBank NP_609051) contains three extended portions that are not seen in mammalian DPP10, DPP6 or DPP4 (the amino acid sequences 265–296, 516–576, and 605–639). They are located within and near the β propeller domain. Based on the crystal structures of human DPP4, DPP6 and DPP10^15^,^16^,^17^, all the three extensions are located at the surface of the protein. The former two extensions are positioned at the connections between β sheets comprising the eight-blade β propeller, whereas the last is at the end of the β propeller domain. The first extension largely consists of alanines and glycines, the second contains a large number of prolines, and the third is rich in charged amino acids. The second extension also exhibits similarity to the proline-rich linker of vinculin, a cytosolic component of the focal adhesion plaque. This region of vinculin is known to interact with several proteins including vinexin, ponsin and Arp2/3^24^,^25^. Therefore, the second extruding peptide in fly DPP10 might mediate the interaction with proteins in the extracellular milieu.

Our previous work demonstrated that the transmembrane and its surrounding region of DPP10 are sufficient for the binding to Kv4 proteins^11^. This portion of fly DPP10 shows high amino acid identity to those of mammalian DPP10 and DPP6, but clearly differs from that of mammalian DPP4 (Fig. 1A). On the other hand, the peptidase domain of fly DPP10 displays similar amino acid identity to mammalian DPP10 and DPP4 (38 and 40%, Fig. 1C). In particular, the catalytic serine and its surrounding area more closely resemble the corresponding portion of DPP4 than that of mammalian DPP10. These sequence characteristics suggest that fly DPP10 might act not only as a Kv4 channel auxiliary subunit, but also as a peptidase.

### Fly DPP10 acts as a Kv4 channel auxiliary subunit

**Figure 2:**
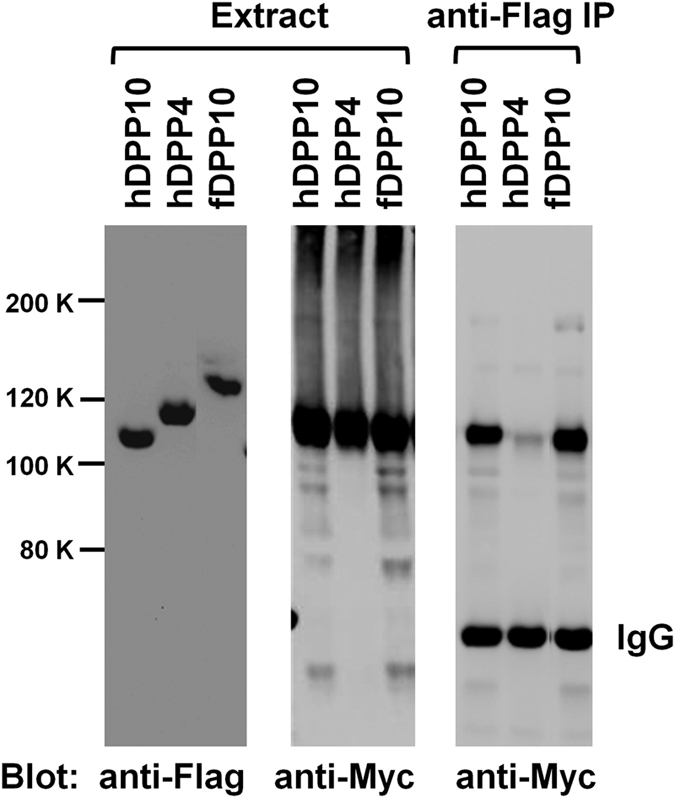
Fly DPP10 binds to Kv4.3 proteins. Triton extracts were prepared from cells transiently transfected with Myc-Kv4.3 and Flag-tagged DPP cDNAs. Extracts and anti-Flag antibody precipitates were examined by immunoblot analysis. Note that anti-Flag antibody effectively coprecipitates Myc-Kv4.3 when it is coexpressed with Flag-hDPP10 or Flag-fDPP10, but not with Flag-hDPP4.

Primary structures of Kv4-family pore-forming proteins are highly conserved across species. The core region of fly Kv4 channel (Shal) polypeptide exhibits over 80% amino acid identity to mammalian channel proteins in this subfamily. Therefore, we used rat Kv4.3 channels to test if fly DPP10 might act as a Kv4 channel ancillary subunit. We first tested if fly DPP10 might form complexes with Kv4 pore-forming subunits by coimmunoprecipitation assays using N-terminally Flag-tagged DPP and Myc-tagged rat Kv4.3 proteins (Fig. 2). Anti-Flag antibody effectively immunoprecipitated tagged fly DPP10, human DPP10, and human DPP4. Importantly, the antibody coprecipitated tagged channel proteins when they were coexpressed with fly or human DPP10, but not with human DPP4. Thus, fly DPP10 tightly binds to rat Kv4.3 channel protein.

**Figure 3:**
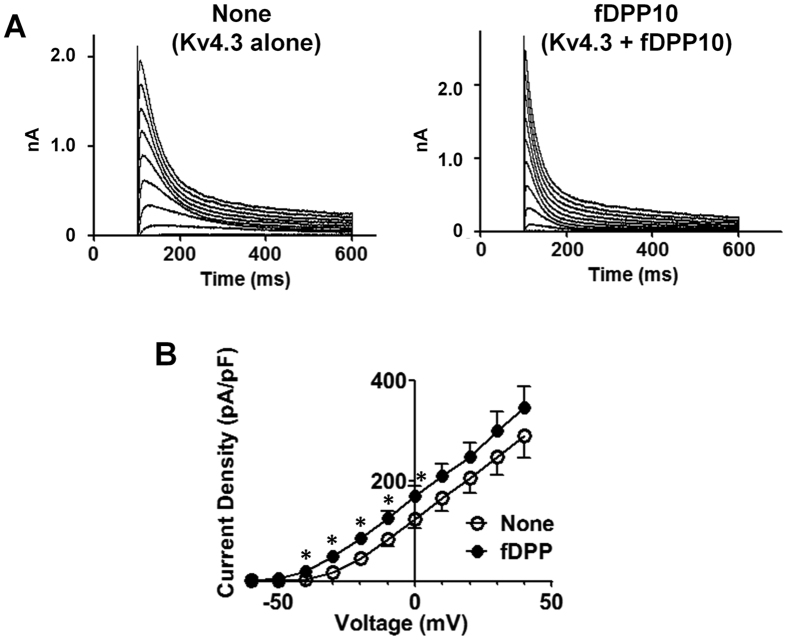
Fly DPP10 increases Kv4.3 current density at low pulse voltages. (**A**) K^+^ currents were measured from cells transiently transfected with Kv4.3 and fly DPP10 cDNAs (fDPP10), or the channel construct and empty vector (None). Currents were elicited by 500-ms square voltage pulses from holding potential of −100 mV. Typical current traces obtained are shown. Note that untransfected cells and cells transfected with fDPP10 alone exhibited voltage-gated outward K^+^ currents with less than 100-pA peak amplitude. These currents in untransfected and fDPP10-transfected cells showed no detectable differences in the amplitude or voltage dependence of activation or inactivation. (**B**) Peak current densities were obtained using cellular capacitance. Voltage-current density relationships are shown (*n* = 13 for None and *n* = 11 for fDPP10). *p < 0.05 compared to None.

Patch clamp recording was then used to examine functional consequences of this complex formation. We transfected 10-times excess fly DPP10 cDNA over rat Kv4.3 construct into HEK293 cells to facilitate complex formation. As expected, voltage pulses elicited large rapidly-inactivating outward K^+^ currents in cells transfected with channel cDNA alone or in combination with fly DPP10 (Fig. 3A). Peak current density was significantly larger with fly DPP10 than channel alone only by pulses to lower voltages from −40 mV to 0 mV (Fig. 3B). The lack of significant raise in current density by more positive voltage pulses suggest that fly DPP10 might not increase the number of functional channels at cell surface. Rather, the fly peptidase homolog might alter channel gating to cause the low pulse voltage-limited enhancement of current density.

**Figure 4:**
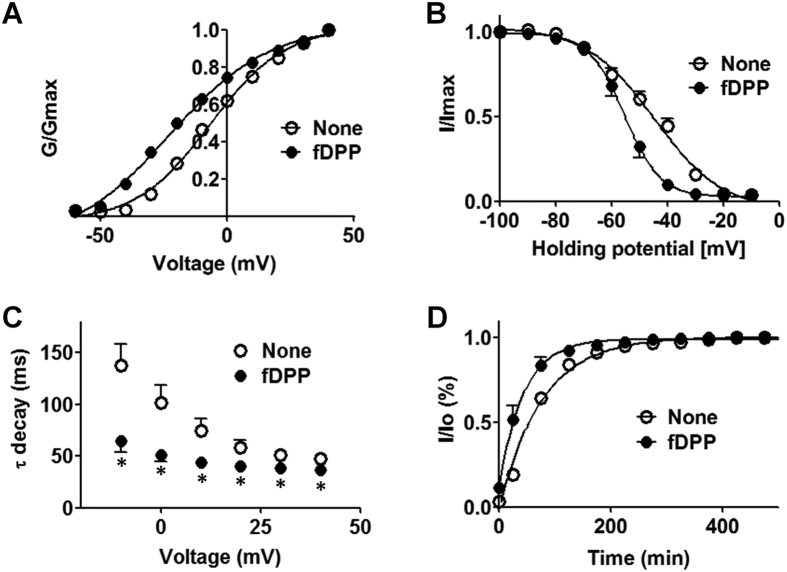
Fly DPP10 alters gating properties of Kv4.3 channel. K^+^ currents from cells transiently transfected with Kv4.3 cDNA in combination with fDPP10 construct (fDPP10) or empty vector (None) were compared for their gating properties. Parameters obtained in this set of figures are listed in Table 1. (**A**) Conductances (*G*) were obtained from the data used in Fig. 1. The relative conductances (*G*/*G*_max_) are plotted against pulse voltage (*n* = 13 for None and *n* = 11 for fDPP10). (**B**) K^+^ currents were elicited by +40-mV pulse from various holding potentials. The ratios of the peak amplitude to that obtained from the holding potential of −100-mV (*I*/*I*_max_) are plotted against holding potential (*n* = 11 for None and *n* = 8 for fDPP10). (**C**) Inactivation kinetics of the data in Fig. 1 was analyzed using one-exponential decay. Obtained time constants are plotted against pulse voltage (*n* = 13 for None and *n* = 11 for fDPP10). *p < 0.05 compared to None. (**D**) K^+^ currents were elicited by two voltage pulses separated by an interval at −100 mV with various lengths. The ratios of the peak current amplitude by the second pulse to that obtained by the first pulse (*I*/*I*_o_) are plotted against interval time (*n* = 5 for None and *n* = 7 for fDPP10).

We therefore analyzed rat Kv4.3 channel gating in the presence and absence of fly DPP10. The fly peptidase homolog markedly shifted a conductance-voltage curve to the negative direction (Fig. 4A). The voltage for half maximal activation was about 15-mV more negative in the presence of fly DPP10 than its absence (Table 1). Likewise, the fly protein affected voltage dependence of steady state inactivation and caused a steeper decline in relative current amplitudes at higher voltages (Fig. 4B, Table 1). Speeding up inactivation is a hallmark of mammalian DPP6/DPP10 actions. Fly DPP10 also influenced inactivation kinetics. Time constants for inactivation estimated by assuming one-phase decay were significantly smaller with fly DPP10 than channel alone at all test pulse voltages (Fig. 4C). Finally, the association with fly DPP10 facilitated recovery from inactivation (Fig. 4D). The time required for half maximal recovery was significantly shorter and about half in the presence of the fly protein than its absence (Table 1). Thus, fly DPP10 markedly alters rat Kv4.3 channel gating, demonstrating its role as a Kv4 channel-ancillary subunit.

### Fly DPP10 exhibits dipeptidyl peptidase activity

**Figure 5:**
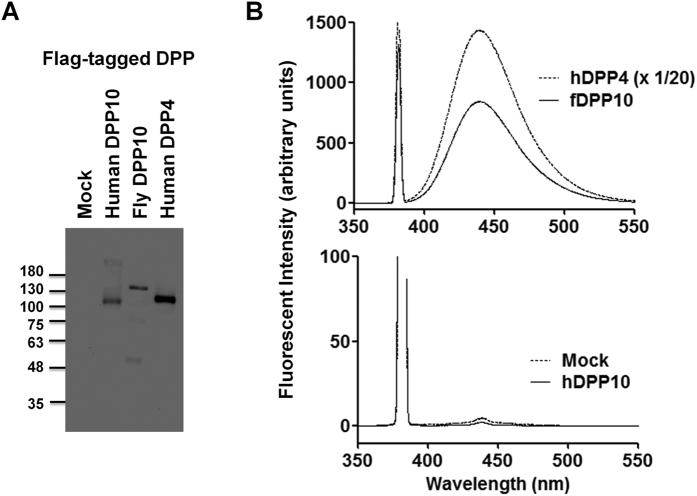
Fly DPP10 exhibits dipeptidyl peptidase activity. (**A**) Triton extracts from cells transiently transfected with Flag-tagged fDPP10, hDPP10 or hDPP4 cDNA, or empty vector (Mock) were analyzed by immunoblot analysis with anti-Flag antibody. When anti-Flag immunoreactivity in the Flag-hDPP4 extract was taken as 1, relative values for the Flag-hDPP10 and Flag-fDPP10 samples were 0.29 and 0.16, respectively. (**B**) The same volume of the prepared extracts, except that the Flag-hDPP4 sample was diluted 20-fold, was used for reaction with 500 μM Gly-Pro-MCA at 37 °C for 30 minutes. After the incubation, the samples were analyzed using fluorescent spectroscopy.

Next we tested if fly DPP10 might possess dipeptidyl peptidase activity. Triton extracts were prepared from HEK293 cells transiently transfected with N-terminally Flag-tagged DPP cDNAs for this purpose. Flag-tagged DPPs were used to compare relative protein expression levels with anti-Flag antibody reactivity. Immunoblot analysis indicated that the prepared extracts contained significant Flag-tagged DPP proteins at the sizes expected from their primary sequences (Fig. 5A). The human DPP4 sample showed strong dipeptidyl peptidase activity with Gly-Pro-MCA as a substrate (Fig. 5B). Similarly, the fly DPP10 extract digested this artificial substrate. In contrast, no activity was detected with the human DPP10 sample. We also determined *Km* and relative *k*cat values for Gly-Pro-MCA under our enzyme assay conditions (Table 2). Fly DPP10 and human DPP4 showed similar *Km* values for this substrate. However, relative *k*cat values obtained by normalizing with anti-Flag immunoreactivity in the extracts significantly differed between fly DPP10 and human DPP4: the normalized *k*cat value with fly DPP10 was about one sixth of that with human DPP4. These results indicate that fly DPP10 possess significant dipeptidyl peptidase activity. They also suggest that the fly DPP10 is less effective than human DPP4 at removing a Gly-Pro from peptides.

### The two functions of fly DPP10 are independent at a molecular complex level

We wonder if the identified two functions of fly DPP10 might interact each other to influence the other activity. We first tested whether the complex formation with Kv4.3 channel might alter fly DPP10’s enzymatic properties. The extract from cells transfected with both fly DPP10 and rat Kv4.3 cDNAs showed *K*m and relative *k*cat values for Gly-Pro-MCA similar to those seen with fly DPP10 alone (Table 2). Therefore, the interaction of fly DPP10 with Kv4.3 channel does not affect its peptidase activity towards this substrate under our assay conditions.

**Figure 6:**
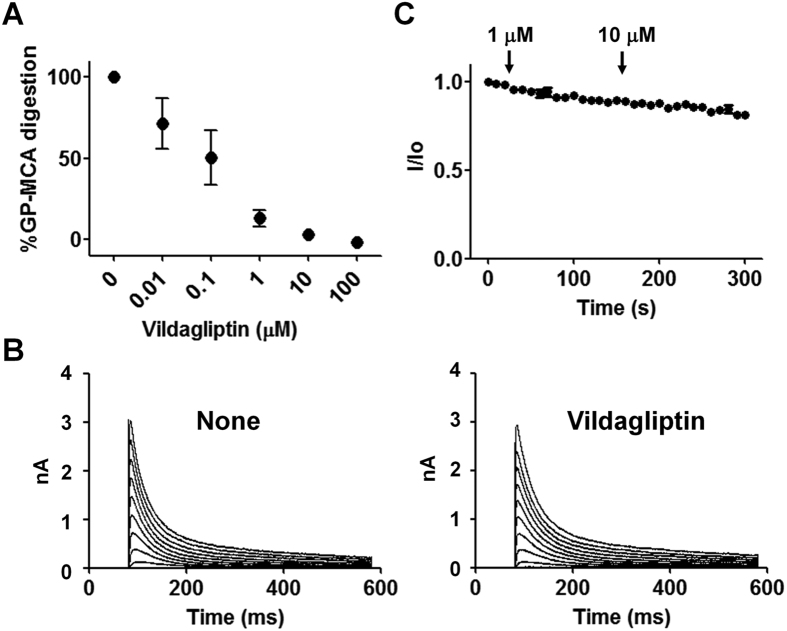
Vildagliptin inhibits the peptidase activity of fly DPP10, but does not influence the gating of the associated Kv4.3 channel. (**A**) Dipeptidyl peptidase activity of Flag-fly DPP10 was determined with Gly-Pro-MCA as a substrate in the presence of indicated concentrations of vildagliptin (*n* = 3). (**B**) The impact of vildagliptin on fly DPP10-Kv4.3 channel complexes was evaluated by perfusing bath solution containing the drug. Top and bottom panels show representative current traces before (None) and 2 minutes after application of 10 μM vildagliptin, respectively. (**C**) Relative peak current amplitudes were measured during perfusion of vildagliptin (*n* = 3). Arrows represent the time at which bath solution was switched to the one containing the indicated concentration of vildagliptin.

Vildagliptin is an anti-diabetic drug that covalently binds to the active site of DPP4^26^. The drug also inhibits other DPP4-related enzymes, such as FAP, DPP8 and DPP9^27^,^28^,^29^. We reasoned that, if the drug inhibits the peptidase activity of fly DPP10, the drug should then covalently modify the catalytic site of fly DPP10. This covalent modification would then be used to test the potential impact of structural changes in the fly DPP10 catalytic portion on the associated channel. We found that vildagliptin effectively inhibits the digestion of Gly-Pro-MCA by fly DPP10, and the half maximal inhibition occurred at a hundred nanomolar range (Fig. 6A). We then examined whether the application of the drug might alter the amplitude or gating of fly DPP10-Kv4.3 channel complexes. The drug at the concentration of 1 or 10 μM produced no significant changes in the current amplitude, voltage dependences of activation, or inactivation kinetics (Fig. 6B, Table s1). Thus, vildaglipin-binding to the active site of fly DPP10 does not influence the expression or gating of the enzyme-asssociated channel.

## Discussion

Pore-forming subunits of ion channels maintain a fairly well-conserved structural architecture. On the contrary, ancillary subunits are highly diverse and might have derived from ancestral proteins with distinct functions during evolution. Mammalian DPP6 and DPP10 are structural homologs of the famous drug target DPP4 that digests physiologically important peptides. However, structural arrangements of the catalytic triad and substrate-recognizing portion are inconsistent with their enzymatic function^16^,^17^. In addition, the attempt to restore peptidase activity of human DPP10 by replacing multiple amino acids including the introduction of serine at the catalytic site failed^17^. These findings suggest that they include more global structural diversions from an enzyme. In this paper, we demonstrated that fly DPP10 is a dual functional molecule, acting as a channel auxiliary subunit and possessing significant dipeptidyl peptidase activity. Our results provide evidence that the functionality of channel ancillary subunits diverge during evolution.

Primary structures of Kv4 channels are highly conserved across species including insects. The amino acid identity between fly and mammalian Kv4 polypeptides is over 70%. This value would be over 80%, when diverse S1–S2 and S3–S4 loops, and C-terminal portions are eliminated. The physiological role of Kv4 channels are also conserved across species. It is established that mammalian Kv4 channels carry dendritic A-type current^30^. This current dampens retrograde spread of excitation and thereby controls integration of synaptic inputs^31^. Using dominant-negative Kv4 subunits, fly Kv4 channel was found to play a similar electrophysiological role in insect neurons^32^. Therefore, it is likely that fly DPP10 forms complexes with Kv4 channel (Shal) to control dendritic A-type current and synaptic signal integration in native insect neurons.

Kvβ subunits are other dual-functional channel-auxiliary proteins. They exhibit aldo-keto reductase activity with NADPH as a cofactor, whereas they form complexes with Kv1 and possibly some other Kv channels^9^,^33^,^34^. As an enzyme, Kvβ proteins show broad substrate specificity and much smaller *k*cat/*Km* values compared to other aldo-keto reductases^35^. Thus, they are ineffective enzymes converting substrates to products. In this study, we found that the *k*cat/*K*m value of fly DPP10 is ~1/6 of that of human DPP4. This somewhat less effective nature may argue against the physiological role of fly DPP10 as a peptide-degrading enzyme. Nevertheless, flies and other insects contain various bioactive peptides not seen in vertebrates. In particular, many tachykinin- and neuropeptide Y-related peptides contain proline at the second position. Thus, fly DPP10 may act as a supplemental enzyme to remove these bioactive peptides, in concert with other dipeptidyl peptidases, such as DPP4. The significant dipeptidase activity of fly DPP10 also supports the possibility that the presence of various important bioactive peptides in flies might provide the pressure to maintain its enzymatic activity during evolution.

Although Kvβ proteins are less likely to act as effective substrate-converting enzymes, their oxidoreductase function may link between cellular redox state and excitability^36^. In this paper, we tested if the channel-regulatory and enzymatic functions of fly DPP10 might interact each other at a molecular complex level. However, complex formation with Kv4 proteins did not affect the enzymatic properties of fly DPP10. Likewise, vildagliptin-binding to fly DPP10 did not influence amplitude or gating of the associated Kv4.3 channel. Although the strategies used to test the possible interactions are certainly associated with limitations, the obtained results suggest that the the two functions of fly DPP10 are independent at a molecular level. The lack of functional interactions in the DPP10-Kv4 channel complexes is further supported by our previous finding that a chimeric DPP protein consisting of the transmembrane portion of DPP10 and the extracellular region of DPP4 is capable of not only forming complexes with Kv4.3 protein, but confers DPP10-induced gating changes in the associated channel^11^. Thus, it is likely that peptidase activity and channel regulation by fly DPP10 independently operate at a molecular complex level. Instead, the two functions may interact at cell or tissue levels under physiological conditions. For example, certain bioactive peptides may regulate Kv4 channel activity upon binding to their receptors. The channel-associated fly DPP10 may remove this regulatory signal at the cell surface, forming a negative feedback loop in this peptide-mediated control of cellular excitability.

Finally, DPP4-related enzymes are considered important therapeutic targets for various diseases. More information on the structures and functions of these molecules will aid the development of safe and effective drugs for target diseases. In this regard, fly DPP10 may be a unique reference placed between the functional DPP4 and non-functional mammalian DPP10.

## Methods

### Complementary DNA constructs

A full-length fly DPP10 cDNA (GenBank #NM_135207) was obtained by several PCRs with an oligo-dT-based cDNA library prepared from adult heads of *Drosophila* melanogaster as a template. Obtained fragments corresponding to various portions of the fly DPP10-coding region were verified by sequencing and connected using endogenous restriction enzyme sites. The connected full-length cDNA was then subcloned into Flag 10 vector (Sigma-Aldrich, St. Louis, MO) using PCR with a primer containing a *Hind*III site in front of the initiation codon. To express the fly DPP10 without a tag, we frame-shifted the introduced *Hind*III site by digesting and filling-in, followed by religation. A human DPP10 and DPP4 constructs were prepared in our previous studies^11^,^37^.

### Immunoprecipitation and immunoblot analysis

HEK293 cells were cultured in Dulbecco’s Modified Eagle Medium supplemented with 10% fetal bovine serum (Gibco, Auckland, N.Z.), 50 U/ml penicillin and 50 μg/ml streptomycin (Nacalai Tesque, Kyoto, Japan) under 5% CO_2_ atmosphere at 37 °C.

For immunoprecipitation, cells on a 100-mm plastic dish were transfected with Flag-tagged DPP cDNA (8 μg) and Myc-tagged Kv4.3 cDNA (2 μg) using the SV40 nuclear localization signal peptide-conjugated polyethyleneimine^38^. Two days after transfection, cell extracts were prepared with the lysis solution containing 20 mM Tris-HCl (pH 7.4), 0.2 M NaCl, 1% Triton X-100, 1 mM EDTA and protease inhibitors. Immunoprecipitation was performed with anti-Flag antibody-conjugated resin, as described previously^39^. Immunoreactive proteins were detected by secondary antibody conjugated with horseradish peroxidase (Jackson Immunoresearch, West Grove, PA) using chemiluminescent reagents (Pierce, Rockford, IL). Chemiluminescence images were captured and analyzed using a CCD camera-based system (UVP, Upland, CA).

### Patch clamp recording

HEK293 cells on a 60-mm dish were transfected with rat Kv4.3 and fly DPP10 cDNAs at the ratio = 1:10 using the peptide-conjugated polyethyleneimine^38^. For Kv4.3 channel alone, empty vector was included instead of fly DPP10 cDNA. We also included a small amount of expression construct for CD8 to visualize transfected cells using anti-CD8 antibody-conjugated beads. Immediately after transfection, cells were reseeded on 35-mm plastic dishes. Two days after transfection, patch clamp recording was performed on cells marked with the beads.

Whole cell voltage-clamp recording was performed with an Axopatch 700A amplifier. Data were acquired and analyzed by a pClamp 8 software (Axon instrument, Union City, CA). The filter was set to −3 dB at 2000 Hz and the P/N protocol was used to subtract leak currents. Whole-cell input capacitance was neutralized directly from the amplifier. Current traces were low-pass filtered by the digital filter of the data acquisition program (pClamp 8). Patch electrodes were fabricated from borosilicate capillary tubing, and had resistances of 3–7 MΩ when filled with the internal solution. Patch pipettes were filled with solution containing (in mM): 140 KCl, 1.0 CaCl_2_, 2.0 MgCl_2_, 11 EGTA, 2.0 ATP and 10 HEPES titrated to pH 7.4 with Tris base. The bath solution containd (in mM): 150 NaCl, 5.0 KCl, 2.5 CaCl_2_, 1.0 MgCl_2_, 10 D-glucose, and 10 HEPES/Tris-base (pH 7.4).

### Enzyme assays

We used extracts from transiently Flag-tagged DPP4- or DPP10-expressing cells for dipeptidyl peptidase assays. Briefly, HEK293 cells were transfected with expression constructs for Flag-fly DPP10, Flag-human DPP10, Flag-human DPP4 or empty vector. Cell extracts were prepared as described for immunoprecipitation except that protease inhibitors were omitted in the lysis solution. Enzyme assays were performed in the lysis solution at 37 °C with Gly-Pro-MCA (glycyl-L-proline 4-methylcoumaryl-7-amide, Peptide Institute, Osaka, Japan) as a substrate. Fluorescent signals were excited at 380 nm and scanned for the emission wave length on a fluorescent spectrophotometer (Hitachi F-7000). The emission light intensity from 400 nm to 450 nm was summed for enzyme activity.

### Statistical analysis

Statistical comparisons between two groups with deviations were performed by Student’s *t*-test with two tails. Multiple comparisons were done by one-way *ANOVA*, followed by the layered *Bonferroni*’s test. For comparing relative *k*cat values, one sample *t* test was used. Data are presented as the mean ± SEM. *p <0.05 and **p < 0.001.

## Additional Information

**How to cite this article**: Shiina, Y. *et al*. Fly DPP10 acts as a channel ancillary subunit and possesses peptidase activity. *Sci. Rep.* **6**, 26290; doi: 10.1038/srep26290 (2016).

## Supplementary Material

Supplementary Information

## Figures and Tables

**Table 1:** Gating properties of Kv4.3 and fly DPP10-Kv4.3 complex channels.

		**None**	**fDPP**	***p*****None vs fDPP**
Voltage dependence of activation	Half maximal activation (mV)	−7.08 ± 0.82 (*n* = 13)	−21.85 ± 1.70 (*n* = 11)	<0.0001
	Slope factor (mV)	14.70 ± 0.54 (*n* = 13)	21.27 ± 2.07 (*n* = 11)	0.0032
Voltage dependence of steady state inactivation	Half maximal inactivation (mV)	−45.20 ± 2.10 (*n* = 11)	−55.41 ± 1.79 (*n* = 8)	0.0028
	Slope factor (mV)	−11.40 ± 1.10 (*n* = 11)	−4.549 ± 1.07 (*n* = 8)	0.0004
Recovery from inactivation	Half maximal recovery (ms)	51.19 ± 2.57 (*n* = 5)	28.04 ± 6.68 (*n* = 7)	0.0191
	Tau (ms)	73.86 ± 3.71 (*n* = 5)	40.45 ± 9.64 (*n* = 7)	0.0190

Electrophysiological parameters were determined as described in the legend for Fig. 4.

**Table 2:** Enzymatic properties of human DPP4, fly DPP10 and fly DPP10 associated with Kv4.3 channel.

	**hDPP4**	**fDPP10**	**fDPP10 ± Kv4.3**
*K*m (mM)	156 ± 53 (*n* = 4)	205 ± 71 (*n* = 4)	268 ± 69 (*n* = 3)
Relative *k*cat	1 (*n* = 3)	0.18 ± 0.02* (*n* = 3)	0.17 ± 0.02* (*n* = 3)

Dipeptidyl peptidase activity was measured with Gly-Pro-MCA as a substrate. The *K*m and *V*max values were determined using the Michaelis-Menten equation. Relative *k*cat values were obtained by dividing the *V*max values by relative anti-Flag antibody immunoreactivities of the samples used for enzyme assays.
