# Supplementary material for: Fly DPP10 acts as a channel ancillary subunit and possesses peptidase activity
Source: Sci Rep. 2016 May 20;6:26290. doi: 10.1038/srep26290 (PMC4873792; doi:10.1038/srep26290)
Supplement: Supplementary Information [file srep26290-s1.pdf]

Supplemental Information

Fly DPP10 acts as a channel ancillary subunit and possesses peptidase activity.

Yohei Shiina, Tomohiro Muto, Zhili Zhang, Ahmad Baihaqie, Takamasa Yoshizawa, Hye-in J. Lee, Eulsoon Park, Shinya Tsukiji and Koichi Takimoto

Table s1 Vildagliptin does not influence gating properties of fly DPP10-Kv4.3 channel complexes

Electrophysiological parameters were determined as described in the legend for Fig. 4 using the data in Fig. 6B. Inactivation time constants were estimated using one exponential decay. None, before perfusion of the drug ; Vildagliptin, 2-3 minutes after perfusion of 10 mM vildagliptin;

		**None**	**Vildagliptin**
Voltage dependence of activation	Half maximal activation (mV)	-19.68 ± 4.42 (*n*=3)	-20.15 ± 4.91 (*n*=3)
	Slope factor (mV)	12.70 ± 4.47 (*n*=3)	11.97 ± 4.90 (*n*=3)
Inactivation time constants	Voltage pulse at 0 mV (ms)	57.20 ± 9.18 (*n*=3)	50.09 ± 12.87 (*n*=3)
	Voltage pulse at +30 mV (ms)	31.88 ± 4.10 (*n*=3)	30.75± 3.71 (*n*=3)
